# Do Private Conservation Activities Match Science-Based Conservation Priorities?

**DOI:** 10.1371/journal.pone.0046429

**Published:** 2012-09-28

**Authors:** Jonathan R. B. Fisher, Benjamin Dills

**Affiliations:** 1 The Nature Conservancy, Worldwide Office, Arlington, Virginia, United States of America; 2 The Elliott School of International Affairs, George Washington University, Washington, D.C., United States of America; Australian Wildlife Conservancy, Australia

## Abstract

**Background:**

Private land conservation is an essential strategy for biodiversity protection in the USA, where half of the federally listed species have at least 80% of their habitat on private lands. We investigated the alignment between private land protection conducted by the world's largest land trust (The Nature Conservancy) and the science driven identification of priority areas for conservation. This represents the first quantitative assessment of the influence of defining priority areas on the land acquisitions of a conservation non-governmental organization (NGO).

**Methodology/Principal Findings:**

The lands acquired by The Nature Conservancy (TNC) were analyzed using GIS to determine to what extent they were in areas defined as priorities for conservation. The spatial analysis of TNC lands was broken up into land known to be acquired in the last five years, five to ten years ago, prior to ten years ago, and anytime during the last sixty years (including previous sets of data plus acquisitions lacking a date). For the entire history of TNC the proportion of TNC lands within the priority areas was 74%. Prior to 10 years ago it was 80%, 5–10 years ago it was 76%, and in the last five years it was 81%. Conservation easements were found to have lower alignment with priority areas (64%) than outright fee simple acquisitions (86%).

**Conclusions/Significance:**

Overall the location of lands acquired was found to be well aligned with the priority areas. Since there was comparable alignment in lands acquired before and after formalized conservation planning had been implemented as a standard operating procedure, this analysis did not find evidence that defining priority areas has influenced land acquisition decisions.

## Introduction

Private land conservation is an essential strategy for biodiversity protection in the USA, where half of the federally listed species have at least 80% of their habitat on private lands [1]. Although land conservation alone may not be sufficient to ensure effective conservation, it is nonetheless an important element of effective conservation [2]–[4]. Given the inadequacy of conservation funding to meet objectives [5], it is important to focus protection efforts on the most critical areas.

The Nature Conservancy (TNC) and other large conservation organizations have invested substantial resources in developing conservation plans intended to guide their decisions about which land areas and bodies of water to conserve. However, despite the investment in developing a scientific method for prioritizing areas for conservation, the degree to which land acquisition actually follows these scientific priorities has not been investigated before now. This analysis represents the first quantitative assessment of the influence of defining priority areas on the land acquisitions of a conservation NGO.

While focusing on priority areas without regard to acquisition and stewardship costs in a given area can lead to a more expensive final strategy [6], [7], taking action in the absence of priority areas may be considerably more inefficient. Underwood et al. found that 90% of observed spending in California was spent in counties that would not be priorities if the goal was to maximize the protection of distinct species at the least cost [8]. Globally, while conservation non-governmental organizations (NGOs) spend more money overall in countries that contain priority areas, priority areas appear to have little influence in determining how money is distributed among the high-priority countries, indicating room for improvement in coordinating prioritization and spending [9].

Prior research has raised the critique that only about 1/3 of conservation assessments actually lead to implementation [10], [11], that they are often overly theoretical (with little thought to the practical details of implementation) [10]–[12], and that delaying action to allow for gathering more data doesn't necessarily lead to more effective conservation decisions [13]. Since many of these assessments are coarse and global, whereas most conservation is local, it is perhaps not surprising that studies have failed to find evidence of plans being followed. However, specific land protection transactions, either by fee simple acquisition (acquisitions that result in TNC being the sole and permanent owner of land) or by conservation easements (also known as conservation covenants), represent a scale at which finer-scale priorities could, in theory, shape action. This is the scale at which we conducted our analyses.

As the largest environmental conservation NGO (by revenue and assets) in the United States [14], The Nature Conservancy's activities have a substantial impact on conservation in the United States. Armsworth et al. (2012) [14] found that of 1,743 nonprofits active in biodiversity conservation that had financial records available, TNC controls more than 25% and 16% of overall assets and revenues, respectively. As both the cost and size of land acquisitions are increasing over time [15], it is increasingly important that these acquisitions are being made using the best available science to guide them. TNC has a complete set of priority areas defined for the United States, as well as a large amount of readily available data on land acquisitions, making TNC a useful case study for examining the impact of defining priority areas on the acquisition of land for conservation.

Prior to developing priority areas, naturalists and field scientists at TNC typically relied on natural heritage program data to assess conservation value before acquisition [Craig Groves, personal communication]. This worked well early on when there were fairly obvious choices for acquisition in terms of habitat or biodiversity, but more subtle characteristics such as complementarity and connectivity were difficult to assess on a case-by-case basis [Edward Game, personal communication]. There were neither pre-existing maps of conservation priorities nor a consistent methodology used across the entire United States to assess such measures of conservation value.

In the mid-1990s, TNC began to implement a methodology for conducting ecoregional assessments and developing a set of priority areas for conservation (also known as the “portfolio” or “ecoregional blueprint”). The priority areas are developed with the intent of representing all relevant biodiversity features in the ecoregion by identifying many individual species, communities, and ecological systems to serve as the targets of planning efforts [16]. The intent is that if protected, the priority areas should represent functional landscapes that ensure the persistence of the conservation targets [16].

The first guidebook on how to carry out these assessments – Designing a Geography of Hope: Guidelines for Ecoregion-Based Conservation – was published in 1997 even as the first ecoregional assessments were already being developed [16]. While the methodology was developed by TNC, it did incorporate several ideas from the published literature and other organizations as it evolved. Key sources of inspiration were work on systematic conservation planning [4] and conserving nature at regional and continental scales [17] [Craig Groves, personal communication]. Many other concepts were incorporated from the literature, including: the necessity of field surveys [18], tools developed for selecting a suite of priority areas [19], [20], the inadequacy of surrogates to represent all species of interest [21], the importance of considering persistence (as opposed to just maximizing current biodiversity) [22], and the need to combine expert-based and algorithm-based approaches [23].

The guidebook was enhanced and updated several times from 2000–2004 [24]–[27], integrated with methods from the World Wildlife Fund (WWF) [28], and further enhanced in 2006 as The Ecoregional Assessment and Biodiversity Vision Toolbox (a website that made the guidelines easily accessible). This methodology also had some influence on the development of the Open Standards for the Practice of Conservation [29], [30], although ecoregional assessments focus primarily on the first two steps of the Open Standards (conceptualize and plan).

According to TNC's Ecoregional Assessment Status Tool (http://east.tnc.org), roughly 2/3 of the ecoregions in the United States completed defining priority areas from 2000 to 2005, and by May 2010 priority areas for the entire U.S. had been defined (although updates and improvements are still being made in some areas).

Our first hypothesis was that overall the acquisition of lands should be well aligned with priority areas on the assumption that TNC chapters base their acquisition decisions on the best available conservation science. We did not expect perfect alignment for several reasons noted in the discussion section. Second, we hypothesized that there would be improvement over time in the match between science-based priorities and land protected by TNC as assessments and planning methods were increasingly formalized and improved. Our third hypothesis was that outright fee simple acquisition of land would show greater alignment with the priority areas than procuring conservation easements. TNC and other NGOs increasingly use conservation easements as a form of protection rather than buying land outright because of cost; fee simple acquisitions on average cost 2–3 times as much per acre as easements [15], [31]. Since more expensive transactions may be scrutinized more closely before the deals are approved, we expected that fee simple land would be more aligned with the priority areas than conservation easements.

Because the total area of the priority areas relative to the total area of a state influences the likelihood of land protection taking place inside a priority area by chance alone, in addition to the simple alignment we also calculated a “science influence score.” This metric measures how much the actual alignment of acquisitions with the priority areas exceeded what would be expected if acquisitions had been made without considering the priority areas across a given state. Although the priority areas were defined by ecoregion, acquisitions are typically made at the state level. Since the investment pattern of TNC acquisitions varies substantially by state [31], [32], we expected to see considerable variation in alignment with the priority areas from state to state as well.

## Materials and Methods

For a more detailed explanation of every step that was taken in this analysis, see Appendix S1. All references to the spatial operations that were performed refer to standard ArcGIS Desktop (10.0) tool names.

Three data layers were used as inputs for this analysis: TNC lands (spatial data for all recorded lands that TNC has a legal interest in), TNC priority areas for conservation, and the 2010 U.S. Census TIGER/Line® State Boundaries. Due to the inclusion of sensitive data in the two TNC data sets, the spatial data is not being published as part of this paper, but the non-sensitive portion of the data (85% of records are public) is available at http://maps.tnc.org.

To ensure data integrity, the repair geometry tool was run at the beginning of the analysis, as well as after each subsequent geoprocessing step. To begin with, each of the three data layers was projected into the Albers Equal Area Conic projection for North America to yield the most accurate area values without having to use different projections for different regions. The priority areas and TNC lands layers were then clipped to match the state boundaries layer in order to eliminate marine and international data, and to allow comparisons of area of each layer to be made to the total area of each state.

Three subsets of the TNC lands data were extracted into separate layers by time period: one contained all entries with acquisition dates prior to January 1^st^, 2000; the next contained all entries with acquisition dates between January 1^st^, 2000 to December 31^st^, 2005; and the third layer contained all entries with acquisition dates from January 1st, 2006 to the date that the data was extracted (July 20, 2011). Note that 57% of the records in the complete TNC lands data set have no transaction date listed; these records are included in the “all TNC lands” layer but excluded from the three subsets that were defined by time period. Two more subsets of the TNC lands data were created: one for conservation easements (where TNC acquired development or other rights, but land ownership remained unchanged), and the other for fee simple acquisitions. Each of the six TNC lands layers (the complete data set, the three subsets of the data by time period, easements, and fee simple lands) was then dissolved to prevent overlapping and redundant entries from being overcounted in area calculations.

The intersect tool was run on the priority areas data and each of the six TNC lands layers, resulting in six new layers that show the areas where the priority areas and TNC lands overlapped for each subset. The union tool was then run with the state boundaries against: each of these six “intersect” layers, the priority areas data, and the six initial TNC lands layers (to allow summaries of the results by state). The final geodatabase containing all layers was converted to a personal geodatabase in Microsoft Access format, where the results were summarized.

Most of the calculations performed on the results are fairly straightforward; for example, to determine the proportion of TNC lands that were in the priority areas we simply divided the area where TNC lands and the priority areas intersect by the total TNC lands area (by state and/or time period as appropriate). The only calculation that is not intuitive is the “science influence score,” which we use to measure how much of an influence the priority areas had on acquisitions beyond what could be expected by chance. Simply examining the proportion of TNC land area within priority areas means that the larger the total extent of the priority areas, the higher the expected alignment would be even if acquisitions were made without considering the priority areas. The science influence score accounts for the total extent of the priority areas, and can be expressed as:
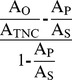
Where:

A_O_ = area of TNC lands that overlap with the priority areas in the state

A_TNC_ = total area of TNC lands in the state

A_P_ = total area of priority areas in the state

A_S_ = total area of the state

Or to rephrase slightly:
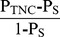
Where:

P_TNC_ = Proportion of TNC land area that overlaps with the priority areas in the state

P_S_ = Proportion of state area that overlaps with the priority areas (i.e. the expected value of P_O_ if acquisitions were to be made without considering the priority areas)

Thus, a science influence score of 0% means that acquisitions were made without any regard to the priority areas (and the alignment shown is what would be expected by chance), a score of 100% means that all acquisitions were in the priority areas, and a score of 50% means that half of the time acquisitions were made without considering the priority areas and the other half of the time they were always in the priority areas.

One important limitation of metrics based on the area of intersection between TNC lands and priority areas is that they do not allow us to assess whether patterns detected were statistically different from random. In order to conduct such an assessment, we reframed the analysis into an examination of outcomes associated with specific events, where each event was a single acquisition. Specifically, we counted the number of records (which corresponds to total number of acquisitions/transactions) where the land acquired had its center (the geometric centroid of the polygon) within a priority area, and compared it to the number of records with their center outside of a priority area. The center was used because the likelihood of any given spatial relationships between two polygon layers is extremely complex but the likelihood of a point being located within a polygon layer is quite simple and also an excellent proxy for finding TNC lands that are mostly within priority areas.

Since there are only two possible outcomes (the center either is or is not within the priority area), we used a chi-square test for goodness of fit with one degree of freedom. The expected number of records within the priority areas under the null hypothesis was equal to (% of US land area covered by the priority area)*(total # of records), or 36.4% * 18,034 = 6,571.

After the initial analysis was complete, we read a random sample of ten ecoregional assessment reports in an attempt to better understand the likelihood of the possible causes of the temporal pattern we found. However, the reports often did not provide sufficient detail about the methods to be certain of the approach taken. As a result, we also contacted several people who were responsible for actually developing the priority areas for ecoregional assessments (generally GIS managers). For the sake of efficiency we reached out to each of the staff members who still work for The Nature Conservancy who was involved in more than one ecoregional assessment (combined they were involved in more than half of all U.S. ERAs). We asked them whether existing TNC lands were preferentially included, excluded, or ignored when selecting priority areas, and for any insight about how priority areas affected land acquisition in their state.

## Results

Overall, more than twice as much TNC land area was within priority areas than would be expected if all acquisition decisions were made without regard to the priority areas. With priority area sites taking up 36% of the total land area of the United States, the fraction of TNC lands within the priority areas was: 80% before 2000, 76% from 2001–2005, and 81% for 2006–2010 [Table 1]. The “science influence score” for acquisitions prior to 2000 was 68%, from 2000–2005 it was 62%, and from 2006–2011 it was 69%. Results were strikingly different between easements and fee simple acquisitions; the science influence score for TNC easements was 44%, while for fee simple acquisitions it was 78% [Table 2].

**Table 1:** Results by time period for all states.

Time Period	Land Acquired, km^2^	% Acquisition Area in Priority areas	Science Influence Score
**All Time**	**41,273.5**	**74.4%**	**59.8%**
2006–2011	3,654.2	80.5%	69.3%
2000–2005	4,244.6	75.8%	62.0%
Pre-2000	7,396.7	79.7%	68.0%
Undated	25,978.0	71.8%	55.7%

**Table 2:** Results by acquisition type for all states.

Acquisition Type	Land Acquired, km^2^	% Acquisition Area in Priority areas	Science Influence Score
Easements	19,898.8	64.2%	43.7%
Fee Simple	5,730.7	86.1%	78.1%

The alignment of acquisitions with the priority areas shows considerable variation from state to state, ranging from a low science influence score of 25% in Pennsylvania to a high science influence score of 97% in Oklahoma [Table 3]. The fact that the science influence score remained positive in all cases means that all states still had stronger alignment of acquisitions with the priority areas than could be expected if they were making decisions without considering the priority areas. This appears to indicate that, to varying degrees, most purchasing decisions tend to either be based on scientifically driven priorities or at least be compatible with them.

**Table 3:** Results by state for all records.

State	Land Acquired, km^2^	% Acquisition Area in Priority areas	% State Area in Priority areas	Science Influence Score
**Total**	**41,273.5**	**74.4%**	**36.4%**	**59.8%**
Alabama	48.7	96.2%	43.4%	93.2%
Alaska	12.4	68.9%	55.3%	30.4%
Arizona	267.6	53.4%	37.2%	25.8%
Arkansas	42.2	47.9%	29.0%	26.7%
California	3,132.0	84.3%	42.0%	73.0%
Colorado	4,367.1	86.0%	50.1%	72.0%
Connecticut	127.7	72.5%	22.8%	64.4%
Delaware	28.3	88.7%	37.7%	81.9%
Florida	357.7	71.9%	55.2%	37.2%
Georgia	185.8	72.5%	23.6%	64.0%
Hawaii	825.4	90.7%	23.1%	88.0%
Idaho	254.2	66.8%	39.7%	44.9%
Illinois	74.2	91.1%	14.9%	89.5%
Indiana	116.8	76.6%	11.8%	73.5%
Iowa	39.5	94.4%	7.7%	94.0%
Kansas	365.1	94.5%	25.1%	92.7%
Kentucky	40.5	75.8%	30.1%	65.4%
Louisiana	45.5	79.8%	34.9%	69.0%
Maine	8,741.9	47.2%	29.6%	25.0%
Maryland	232.8	77.3%	18.2%	72.2%
Massachusetts	77.0	84.8%	30.8%	78.1%
Michigan	226.5	89.3%	20.1%	86.6%
Minnesota	316.6	84.9%	31.6%	77.9%
Mississippi	54.3	66.1%	47.3%	35.8%
Missouri	117.8	94.8%	11.3%	94.1%
Montana	1,655.9	67.9%	31.6%	53.1%
Nebraska	390.7	82.8%	53.2%	63.3%
Nevada	23.3	96.4%	32.9%	94.6%
New Hampshire	332.2	96.2%	46.3%	92.9%
New Jersey	95.5	80.5%	33.5%	70.7%
New Mexico	3,495.4	81.6%	38.1%	70.2%
New York	2,708.6	74.0%	30.2%	62.8%
North Carolina	4,256.4	93.0%	39.3%	88.5%
North Dakota	91.0	94.5%	6.9%	94.0%
Ohio	86.5	97.6%	18.1%	97.0%
Oklahoma	305.2	98.2%	31.8%	97.3%
Oregon	394.4	89.9%	36.2%	84.2%
Pennsylvania	159.8	35.5%	14.3%	24.8%
Rhode Island	85.9	61.4%	22.7%	50.1%
South Carolina	663.5	93.3%	34.5%	89.8%
South Dakota	295.1	88.8%	16.2%	86.7%
Tennessee	72.7	86.0%	48.8%	72.8%
Texas	1,699.8	84.3%	31.0%	77.3%
Utah	224.1	67.3%	44.8%	40.6%
Vermont	316.1	60.8%	29.9%	44.0%
Virginia	1,134.4	81.9%	28.0%	74.9%
Washington	251.5	70.1%	37.9%	51.9%
West Virginia	356.2	90.3%	29.8%	86.1%
Wisconsin	140.2	96.3%	38.9%	93.9%
Wyoming	1,941.4	60.7%	31.3%	42.8%

When we examined alignment with priority areas in terms of the number of acquisitions rather than by the total overlapping area, we found 14,739 records for TNC lands with their center within a priority area, and 3,295 records with their center outside of a priority area. Under the null hypothesis we would expect 6,571 records with center in the priority areas, and 11,463 records outside of priority areas. Thus there was a highly significant positive relationship between the location of TNC lands and the priority areas (χ^2^ = 15,976, 1 d.f., p<0.0001).

Of a random sample of ten ecoregional assessment (ERA) reports (out of 72 available in the United States), two appeared to be weighted in favor of including TNC lands in priority areas, one appeared to be weighted against including TNC lands, one appeared to have ignored protection status, and the remaining six were unclear. Of the four reports that mentioned protected areas in regards to the creation of priority areas, only one explicitly mentioned TNC lands.

The results of asking staff members about the process they used when developing priority areas revealed divergence in the approaches taken, but some common trends. See Appendix S2 for the full text of the responses received. Most respondents indicated that existing TNC lands were incorporated into priority areas in one way or another. In many cases spatial data was unavailable, but during expert review most or all TNC lands were considered. Most of the time TNC lands were weighted towards being included, but not automatically included. However, sometimes (although not as often) TNC lands were not considered, or deliberately excluded from the priority areas. Finally, in some assessments natural heritage program scorecards (which were often used to guide acquisitions before ecoregional assessments were conducted) were automatically included in priority areas, which would lead to the same result as including most TNC lands directly in priority areas.

## Discussion

In interpreting the results, it is important to first consider what level of alignment between TNC lands and priority areas is reasonable and desirable. It is not expected that the alignment would approach 100% even after the priority areas were delineated for four reasons. The first is that TNC is a federated organization, with each state office making its own independent acquisition decisions with the priority areas serving only as guides rather than firm prescriptions. Differences in the size, resources, and leadership of each state program leads to variety in the degree to which they adopt the priority areas versus making their own decisions. Second, acquisitions which are not entirely aligned with priority areas sometimes offer unique opportunities to acquire land at an unusual scale and/or price (including donated lands), and as such may be able to meet goals more efficiently than land within the priority areas. Third, land parcels may have substantial overlap with priority areas but go beyond them as well. Rather than rejecting or reselling the edges of the property which occur beyond the priority area, it is typically more beneficial to keep the larger, more intact parcel. Finally, as new information becomes available about the location and habitat requirements of species of concern, we may find that over time the priority areas no longer reflect the best available science. As a result of these issues, while a high level of alignment is desirable, 100% overlap would be not only unrealistic, but undesirable if it prevented us from being flexible enough to achieve our goals in the most efficient manner.

One striking finding emerging from this analysis is the high degree to which TNC land acquisition has taken place in areas identified by TNC's scientists as priorities for conservation. However, we were surprised that there is no evidence that defining priority areas directly led to focusing land acquisition within those areas over time. Indeed, even before systematic ecoregional assessments had been implemented, land acquisitions tended to be in areas later identified as priority areas by formal planning exercises. There appear to be three likely explanations of this result: priority areas may have been more often defined on existing TNC lands, the same data could be underlying acquisition decisions made before and after the priority areas were defined, and the priority areas could simply have not influenced acquisition at TNC.

Many ecoregional assessments either automatically included TNC lands in the set of priority areas [33], or heavily weighted them for inclusion (as noted in the results section). This almost certainly artificially inflated the alignment between priority areas and older acquisitions; in an ecoregion where 100% of TNC lands became priority areas it is likely that alignment would *decrease* over time (relative to 100% alignment) after the priority areas were defined. While most of the ecoregional assessment reports do not make it clear how widespread this was, discussions with the staff involved in producing the majority of the priority areas indicate that the majority of ecoregional assessments *did* favor including TNC lands in the priority areas. This means that if the priority areas had been developed without favoring existing TNC lands, overlap should have been lower for the lands acquired before 2000, and the priority areas may in fact have influenced acquisitions.

For the second explanation, since lands acquired before ERAs were complete were often based on natural heritage program scorecards, and those scorecards were used to inform the priority areas, some of the correlation is due to the same data source being used for both [Craig Groves, personal communication]. In other words, in the absence of a formal evaluation process, TNC staff still managed to get enough data to lead them to the “best” places to acquire in terms of conservation value. This would indicate that the formal ecoregional assessment process may not have been necessary to determine where to acquire land, although it is also possible that earlier land acquisition decisions were easier to make (when less land had been protected there may have been more obviously “special” areas to focus on protecting) [Edward Game, personal communication].

Third, it could simply be that as others have speculated [10], [12], conservation practitioners (those who make the land deals) are not significantly influenced by science-based priorities. In at least some states parcels were studied on their own and only compared to the priority areas later (where overlap was considered a “bonus”) [Gen Green, personal communication]. Discussions with land protection staff at TNC reveal significant variation in their attitudes about the value of the priority areas in guiding acquisitions. Even if staff attempted to use priority areas to determine acquisitions, low willingness-to-sell among landowners may have made it impractical to consistently do so [34]. However, the overall high alignment with acquisitions and priority areas indicate that even if the priority areas aren't directly influencing acquisitions, the staff acquiring land likely have a good understanding of important areas for biodiversity. While relying solely on experts to define priority areas can lead to ineffective and inefficient outcomes [23], it appears that TNC's acquisitions are mostly compatible with biodiversity objectives.

It is likely that each of these three factors contributed to the result shown, although we believe the first one (heavy inclusion of existing TNC lands in priority areas) is likely dominant based on discussions with the staff who delineated most of the priority areas. A precise quantitative determination is difficult to make given the age of the ERAs (and lack of detail about the methods they used), staff turnover, and the size of The Nature Conservancy. Further investigation is needed to answer the fundamental question of how much influence defining priority areas had on acquisitions at The Nature Conservancy.

The sharp differences in alignment with science priorities between easements and fee simple acquisitions and between states also warrants further investigation. Easements commonly are partially donated and are consistently much less expensive than fee simple land acquisition [15], [31]. It is possible that this lower cost leads to less consideration of alignment with priority areas; more expensive purchases may be examined more closely to ensure they are worth the investment. The large variation in alignment with priority areas between states is less easily explained. Science capacity varies among TNC programs, which may contribute to the variation. Alternatively, since willing sellers are necessary for private land conservation [34], in some states opportunities may be so limited that conservation acquisitions have to be in lower priority locations. Other states may have so few natural areas left that alignment with priority areas is inevitable.

There are a number of problems with the data (e.g. 57% of the records for TNC lands don't have an acquisition date available) which are more fully explained in Appendix S3. These problems reduce our confidence in the results and make further analysis challenging. Since land acquisition remains the strategy for which TNC is best known, it behooves the organization to devote additional resources to making this data more complete and consistent so that they can more confidently display and analyze their acquisitions.

## Supporting Information

Appendix S1 **Detailed Spatial Analysis Methodology.** See Appendix S1-CompleteMethodology.docx.
(DOCX)

Appendix S2 **Personal Communications.** See Appendix S2-PersonalCommunications.docx.
(DOCX)

Appendix S3 **Data Errors and Limitations.** See Appendix S3-DataErrorsandLimitations.docx.
(DOCX)
