# Supplementary material for: Do Private Conservation Activities Match Science-Based Conservation Priorities?
Source: PLoS One. 2012 Sep 28;7(9):e46429. doi: 10.1371/journal.pone.0046429 (PMC3460893; doi:10.1371/journal.pone.0046429)
Supplement: Appendix S1 — Detailed Spatial Analysis Methodology. See Appendix S1-CompleteMethodology.docx. (DOCX) [file pone.0046429.s001.docx]

**Methodology**

Note that ArcGIS Desktop 10.0 was used for this entire analysis, and all references to tools and spatial operations run refer to the standard names for the tools in ArcGIS Desktop.

**Acquiring the Base Layers**

The following three layers were used for this project:

1. TNC Lands (all recorded lands where The Nature Conservancy has a legal interest)

2. TNC Priority areas

3. The U.S. Census TIGER/Line® (State Boundaries) Shapefile for 2010

Public versions of the first two layers above (with sensitive records filtered out) can be acquired from TNC's website: <http://maps.tnc.org/index.html>. TNC staff can download the complete data from <https://maps.tnc.org/coredatainternal/>. If you have any questions about acquiring or using these layers, please e-mail [core_data@tnc.org](mailto:core_data@tnc.org) for more information. The state boundaries layer can be acquired from <http://www.census.gov/geo/www/tiger/shp.html>. It is a shapefile for the entire United States, divided by state, and includes territorial waters.

Due to periodic updates to each of the layers above since this project was initiated, an independent reproduction of this analysis using the most current data available would likely yield slightly different results.

**Projection into Albers Equal Area**

The first step was to take the above layers and reproject them into the North American Albers Equal Area Conic projection (so that we would be able to calculate the area of the various derived layers later on). This projection was chosen to yield the most accurate areas across the entire United States without having to use multiple projections for different areas. The layers from TNC were originally in the GCS WGS 1984 coordinate system and datum, and were converted into the GCS North American 1983 coordinate system. The NAD_1983_To_WGS_1984_5 transformation was used. After this was done, the geometries of all layers were repaired (the repair geometry step took place at the end of each step as a precaution).

**Elimination of Overlapping Features**

Each of the data sets have overlapping features, but when calculating area we want to eliminate any overlap within each data set to prevent over counting. For the priority areas this often occurs at the boundaries of each ecoregion; typically when defining priority areas they were not clipped to each ecoregion. For TNC lands this often occurs when different property rights are tracked for a single property. Both TNC layers (the priority areas and TNC lands) were dissolved in order to eliminate the overlapping features.

**Focusing on the United States**

Using the TIGER/Line® Shapefile as a base, the conservation priority areas layer and the TNC lands layer were both clipped to the United States, eliminating all areas outside of terrestrial U.S. territory. Once again the geometry of each layer was repaired.

**Creating Subsets of the TNC Lands Layer**

Three additional subsets of the TNC lands layer were created in addition to the complete layer to allow analysis by time period. The field used to determine the date of purchase was “CLS transaction date”; CLS stands for Conservation Lands System and is TNC’s official legal database of record for land purchases. One of the new layers contained all entries with CLS transaction dates prior to January 1^st^, 2000; the next contained all entries with CLS transaction dates between January 1^st^, 2000 to December 31^st^, 2005; and the third layer contained all entries with CLS transaction dates from January 1st, 2006 to the date that the data was extracted (July 20, 2011).

Two more subsets of the TNC lands data were created: one for conservation easements (where TNC purchased development or other rights, but land ownership remained unchanged), and the other for fee-simple purchases (where TNC purchased the land outright) using the “Protection Mechanism” field. Each of the six TNC lands layers (the complete data set, the three subsets of the data by time period, easements, and fee-simple lands) was then dissolved (eliminating overlapping and redundant entries) and the geometries of all four layers were repaired. Many records in the data did not have any CLS transaction date record; these entries were left in the “all TNC lands” layer but were excluded from the subsets of the data by time period.

**Intersections of Priority areas with TNC Lands**

The intersect tool was run on the priority areas data and each of the six TNC lands layers, resulting in six new layers that show the areas where the priority areas and TNC lands overlapped for each subset. The geometries of these six results layers were then repaired.

**Union with TIGER/Line® (State Boundaries) Shapefile**

A union operation was then run on the TIGER/Line® state boundaries shapefile and each of the seven base layers (priority areas and the six TNC lands layers) as well as the six results layers, so that we could summarize each layer by state. Each of the resulting 13 “unioned” layers was then repaired one last time, and the state backgrounds were deleted from each of the final layers.

**Final Calculations**

The entire file geodatabase was then converted to a Microsoft Access personal geodatabase. The shape_area column was used as the source for the area of each record (it was stored in m^2^, but converted to km^2^ for reporting purposes), and a few calculate geometry operations were run to verify that this value was correct. The area of each layer was then summarized using SQL queries in Access, yielding the total area for each layer by state and for the entire U.S.

**Transaction Analysis**

### To calculate the number of TNC Lands records that had their centroid within the priority areas, we used the Select By Location tool. We used TNC Lands as the target layer, the priority areas as the source layer, and the spatial selection method of “Target layer(s) features have their centroid in the Source layer feature.” This returned the number of records with their centroid in the priority areas, we used the “switch selection” button to get the number of records with their centroid not in the priority areas.

### Excel was used to calculate the p-value for the chi-square test.
