# Supplementary material for: Do Private Conservation Activities Match Science-Based Conservation Priorities?
Source: PLoS One. 2012 Sep 28;7(9):e46429. doi: 10.1371/journal.pone.0046429 (PMC3460893; doi:10.1371/journal.pone.0046429)
Supplement: Appendix S2 — Personal Communications. See Appendix S2-PersonalCommunications.docx. (DOCX) [file pone.0046429.s002.docx]

Personal Communications

The following set of people all worked on ecoregional assessments (ERAs); with one exception (which is noted below) they were GIS managers who were the ones creating the layer of priority areas (AKA ecoregional portfolio). They were asked this question: “*In your experience how often were existing TNC lands included when creating an ecoregional portfolio? Are they always included, usually left out (since they're "already conserved"), or somewhere in-between?*” The responses were as follows:

1. Jan Slaats, 8/15/12 (involved in 5 ERAs, deeply involved in one):

I was only deeply involved in one ERA: Superior Mixed Forest. I didn’t work on other Ecoregion’s planning projects that were done out of this office, since there were other staff who led those at the time.

But for the SMF, I don’t think that the existing TNC lands had much of an influence on the portfolio development, if any, simply because TNC didn’t have very many preserves in that Ecoregion.

Especially in MN, the focus had been on the Northern Tallgrass Prairie Ecoregion’s preservation, in part because that was one of the first Ecoregions to undergo an ERA.

1. John Prince, 8/15/2012 (involved in ~17 ERAs):

I was involved, to various degrees, in most of the Ecoregional Assessments in the Southeast U.S. Those that I had more involvement in and those where I was the primary GIS person on the team followed a similar methodology which I’ll describe here.

For a given ecoregion a planning team was convened and several subteams (mostly for target groups - plants, animals, natural communities) were formed. A list of conservation targets were agreed upon, goals for the targets were set, and occurrences of those targets were assessed/prioritized. Mostly, the target occurrences were prioritized by quality and availability toward meeting target goals. For example; a lower quality target occurrence may be given a high priority if there were few occurrences of that target to go toward meeting the goals while if a specific target had many occurrences to choose from only the higher quality ones would be given priority.

These portfolio assembly meetings usually consisted of hardcopy maps (usually at 1:250,000 scale) that showed the prioritized target occurrences on landcover with protected areas and other ancillary data such as roads, political boundaries, water bodies. We did include TNC ownership when we had it but back then that was not always available. The experts at the portfolio assembly meeting were well acquainted with the landscape and included TNC staff from the states involved in the ecoregion; and therefore, they mostly knew where the TNC properties and lands TNC was interested in were located. When drawing the portfolio boundaries on the map they usually (or always) drew those properties into the portfolio sites (which often had relevant target occurrences in them anyway). After the meeting the experts had opportunities to check the digitized boundary with what they intended to capture and make edits before finalizing the portfolio.

1. Mary Harkness, 8/5/2012 (involved in ~12 ERAs):

So in the early days - and even in the middle days of TNC ecoregional planning - we so often didn't have the spatial data that showed the TNC preserve boundaries. At least in the Midwest and Great Plains, a lot of the ERAs were based on a combination of qualitative assessments (with the numeric conservation goals) of EO data and vegetation data, with expert input. So, even though spatial data for TNC lands was generally not readily available (I was shocked by this when I started there back in 1997, fresh out of grad school :) ), I think the expert input was such that TNC lands got included. Trying to remember - in very early ones that others worked on in Midwest (maybe NTP), I think preserved and managed lands were looked at as a starting point for the overall ERA - because they were already at least partially conserved, if not fully. But I don't think that was normally the case for those ecoregions. In ones I worked on, there was definitely an attempt to look at things from the ground up, regardless of ownership. Also in the Midwest and Great Plains, the ecoregions are so highly converted, that even if you didn't include ownership considerations / TNC preserves, they would have usually come out in the mix anyway. I think in the MW and GP, it wasn't necessarily usually conscious/intentional - but on the other hand, there's no way that a significant preserve would have been left off. actually, I'm remembering now in Michigan in the Great Lakes, how there was a lot of debate around a few much smaller preserves along the east side of Lake Michigan - and I think they were either considered low priority, and maybe one or two didn't even make it in?  I think the team caved and let them in, but as low priority sites.

 Another thought - given relationship/origins of Heritage programs and TNC, and TNC use of Heritage EO data to inform land protection efforts and later ecoregional planning - and that in early days TNC was trying to preserve areas with good EOS...I think it's really intertwined and hard to tease out.  Again, not necessarily a specified part of the planning process, but it would be surprising in MW and GP if any of the major TNC preserves/projects were left out - it just wouldn't have happened.

1. Tyrone Guthrie, 8/3/2012 (involved in about 12 ERAs):

Generally protected lands (incl. TNC) would have been used in the cost surface if the ERA used a MARXAN type process [meaning they weren’t automatically included but were weighted towards inclusion]. If connectivity was then included as a driving input, they would be more likely to be part of a portfolio site.  A similar subjective process may have happened with the older expert delineated plans.  Each plan is different, of course, in how they prioritize the input factors, however your results would be what I'd expect.

1. Craig Groves, 7/11/12 (developed TNC’s ERA process and was involved in several ERAs, although not as a GIS manager):

I have some pretty good ideas of why it turned out that way and some ideas for further analysis.  Just one example – before ecoregional assessments, state programs based land acquisition on natural heritage program scorecards. In many cases, the areas highlighted in these scorecards were 100% incorporated into ecoregional assessment priorities – so that would drive the high correlation before –after in part. Since far more of our conservation work is about easements, I think it is equally important to note how poorly that match is and figure out why. Hope to chat at SCB. The good news is that we focus our acquisitions to a large degree on priority places.  Also of note – at one point around 2000 John Sawhill put a board policy in place that any project over $1 million had to fall within a portfolio of sites, and there were no exceptions for several years.

1. Gen Green, 3/13/2012 (involved in 2 ERAs):

My feeling is that the relationship between TNC owned or managed lands and portfolio selections here is that both types have threatened biodiversity features. Portfolio sites are so large and broad that individual acquisition seems to have little to do with the fact that it is also a portfolio site. When deciding to pursue an acquisition the individual parcel is studied for its contribution to conservation rather than giving it a thumbs up based on ERA. If its also in a portfolio site that is an added bonus. I seem to recall the Utah High Plateaus ecoregion placed existing TNC lands in a category with other protected areas (National Parks etc) so that the portfolio selection process would look elsewhere to met goals. I don’t recall about the other plans in Utah.

1. Holly Copeland, 3/13/2012 (involved in 3 US ERAs):

In Wyoming the Utah-Wyoming Rockies plan seeded in preserves and protected areas. To the best of my knowledge, all other plans intersecting Wyoming did not. I suspect that some congruence is due to, as you say, deliberately including those preserves in the portfolio (especially  in our earlier plans – Wyoming basins), and some congruence is due to a gut feeling that the area was important, as that was born out by a Marxan analysis. It would be difficult to sort it all out, and would require a case by case assessment, I think, to really know. I hope this helps.

1. Michael Schindel, 3/13/2012 (involved in 4 ERAs):

Until 2007 we locked in all AUs [analysis units used when developing the priority areas] with more than ~ 10% protected lands in our Marxan scenarios. We stopped that during the Cascades EA as we found that the extensive protected areas in the upper elevations were skewing our results. We didn’t lock in anything, but then added those protected AUs back in post analysis.

To your question more specifically, my general sense is that most TNC preserves are acquired to protect local resources that may not necessarily reflect regional priorities. Also, many of the preserves were acquired before ecoregional assessments were done. It caused a little heartburn here in Oregon when it was noticed that one of our biggest preserves, Juniper Hills, was not selected in the base Marxan scenarios. I suspect each chapter has responded differently to this sort of situation, but I would bet that most “burned in” all preserves and other protected lands after the fact if they weren’t locked in up front. That would mess with your central premise, unless you’re restricting your data inputs to those tracts acquired after a portfolio was defined.

1. Leo Sotomayor, 3/13/2012 (involved in 1 US ERA, many Latin American ones):

From the beginning of portfolio definition in MARXAN type tools, it was common to seed the optimization tool with existing TNC sites and protected areas. This was not mandatory, but rather left up to each team. Some did include them and some did not, hard to say which strategy is better or was used by more people overall… I would take a quick read of the portfolio definition methods in each Ecoregional plan to get a better idea. In some ecoregions, the choices may be limited, so you may see more congruence with TNC lands because of this as well, but in others there may be more options and planners may have wanted to go beyond what was already conserved…

In Latin America, most of the ecoregional plans seeded their portfolios with existing public Protected Areas, since there was so much work in protected areas already by TNC and partners, we wanted to keep this investment. Other analyses, such as country level GAP analysis, did not include protected areas, as the objective there was to find areas of high biodiversity that did not have any protection.

In addition to asking GIS managers about the process of developing the priority areas, Adrianne Burk (Director of Conservation Information Management at The Nature Conservancy, which is the group that manages the information relating to TNC’s legal interests in lands) was consulted on two questions:

1. Adrianne Burk, 8/3/2012 (in response to “*Can you confirm the following statement? In CLS we can track which of our acquisitions were purchased and which were donated, but we do NOT know for the lands donated whether we approached the owner (targeted acquisition) or whether the owner approached us (opportunistic acquisition).*”):

No, we can’t differentiate how the initial contact was made.  However, if TNC is offered a parcel out of the blue that does not fit our mission, we aren’t supposed to accept.  Pretty much, OUs follow that rule.  I know most OUs will usually suggest other orgs or gov agencies that may be interested (e.g., a local land trust).  Most OU staff are aware of what areas may be of interest to gov agencies and other orgs, and if the parcel is in such an area, the OU will try to effect a protection action (sale, gift, whatever), including doing an Assist or even a pass-through where TNC is in the chain of title.

Of course, there are major donor situations and other internal political situations when an OU will take on an out-of-the-blue parcel.  But unless we can turn it over fairly quickly or convince the donor it’s trade land, most OUs don’t do it.  In fact, I know of parcels granted to TNC in wills that TNC has legally not accepted.  I have no idea what the executor of the will does in such a case – probably sell it – but it does happen.

The above is from my own experience with friends/family/vague acquaintances who have asked me about selling/donating land to TNC.  If you need further information, I’ll think of someone else to contact.  *Adrianne*

1. Adrianne Burk, 4/17/2012 (in response to *“I know we regularly transfer our land to the government, but I'm wondering if we ever do the reverse (or have done so in the past) where the government transfers land to us?”*):

Yes, we do have transactions from gov units to TNC.  For several western major projects, we did do land exchanges where we transferred conservation land to gov and gov transferred to TNC “trade land” parcels, e.g., we transferred some land in TX to the Forest Service, and the USFS transferred some pine plantations to TNC, which TNC sold as trade land.  We’ve done such land exchanges elsewhere but I am most familiar with lands west of the Mississippi.

Other times, we’ve simply bought straight out some gov land, e.g., from BLM to add to a preserve. I want to add that gov land to TNC is insignificant compared to TNC land to gov, or even TNC Assist land to gov.  Not to mention other land protection activity.

Finally, one of our early reviewers had an important insight. He was not involved in TNC’s original ERAs, but he has worked extensively on updating the ERA methodology at TNC and has interviewed many people who were more directly involved.

1. Edward Game, 4/13/2012:

I think some nuance on this point [that an informal land selection process was confirmed by a more rigorous ERA later] is that in each state the first places we went were fairly obvious choices, distinctive places – so we were pretty good at picking the same sort of places that would inevitably be included, whereas later on we were targeting complementarity in the portfolio, something people are much poorer at picking intuitively.
