# Supplementary material for: Do Private Conservation Activities Match Science-Based Conservation Priorities?
Source: PLoS One. 2012 Sep 28;7(9):e46429. doi: 10.1371/journal.pone.0046429 (PMC3460893; doi:10.1371/journal.pone.0046429)
Supplement: Appendix S3 — Data Errors and Limitations. See Appendix S3-DataErrorsandLimitations.docx. (DOCX) [file pone.0046429.s003.docx]

Appendix 2 – Data Problems / Limitations

It should be noted that there are a number of known errors and limitations in the data sets used in this analysis. First, although TNC has been buying land for 60 years, since the portfolio was mostly developed between 2000 and 2005, it is only in the last five years of purchases that the complete portfolio was available to have an impact on purchasing decisions. Between 2000 and 2005 some purchases could have been guided by priority areas which were defined earlier, but as noted above the higher alignment in purchases before 2000 is most likely due to the portfolio being defined to include existing properties rather than the other way around.

For the TNC lands data itself, there is missing data (there are some shapes missing entirely, 56% of all records are missing dates, etc.) as well as some erroneous data (e.g. duplicate shapes for the same parcel). There is also some inconsistency in how lands we no longer own are tracked (the data includes some historical data, but not all of it). Finally, the spatial data does not include any purchases which TNC facilitated for a partner organization, and mostly does not include lands which we used to own but which have been transferred to a partner organization since. While we continue to work on improving the data, it is complicated by a lack of an organizational mandate to provide it, a federated organization structure where state chapters are fairly autonomous (and use very different data models), and other GIS projects competing for staff time. Prior to 2008 TNC only had spatial data available for easements, and the inclusion of fee-simple properties has been a major undertaking.

Overall the spatial layer used for TNC lands consisted of 41,274 km^2^, indicating that our spatial data is in-between the current holdings (fee-simple ownership and easements) according to the official legal database of 22,129 km^2^, and the total cumulative purchase of 61,308 km^2^ which includes lands transferred to the government or other partners.

In an effort to better understand some of the problems with the TNC lands data, and to determine whether or not certain states or types of records should be excluded entirely, we looked at three metrics: how many records were missing IDs (which provide a link to TNC’s legal database with much more details about each purchase), how different the total purchase area in our layer was from the official figures for current holdings, and how much of the data had no date (in almost all cases a lack in date was due to a lack in an ID).

On their own, none of these metrics inherently indicates that the data is erroneous. A difference in the area figures between the spatial data and the official figures could indicate that the spatial data still includes TNC purchases which have since been transferred to other partners (which as past TNC purchases are still valid for this analysis). A missing date or ID means that we cannot easily find out more information about a given parcel, but it does not necessarily indicate the data should be ignored either (it may or may not be valid). In some states TNC staff have indicated that the IDs are missing (especially for older records) because they were not required to enter them, but that they carefully update the spatial data to ensure that they are an accurate depiction of our current legal holdings.

Since each of potential indicators of error we examined (area discrepancy between the spatial data and legal database, or area discrepancy relative to total state area, or lots of missing dates or IDs) were insufficient on their own to decide if data is faulty or not, we decided to see if they were correlated. Each factor was compared against each of the others in a scatterplot, and no relationship was found. The linear trend lines for each plot had an R^2^ value of between 0.0002 and 0.0179; the only correlation was between records missing IDs and records missing dates, but since all records with valid IDs have dates assigned during quality control, this was expected. If they had been correlated, a stronger case could have been made that some of the records were likely erroneous and should have been excluded, but as is there was no easy way to identify which states had mostly “bad data.”

Given some of the problems with the data (described at length in the discussion section), we separately analyzed the 14 states with the best data (>95% of the area had a date, no missing IDs, and area discrepancies of less than 100 acres or 0.4 km^2^). They had higher alignment (84% for all records) and science influence scores (75% for all records) than the entire data set [Table3], but this is likely due in part to the fact that they are mostly Midwestern states where little natural areas exist outside of the portfolio due to conversion to agriculture.

We have identified three possible areas for future analysis. First, when we calculate our science influence score, we effectively assume that all land in the state is potentially available for purchase. If we were to exclude land converted to non-natural cover (e.g. urban or agriculture) as well as public land which is typically not for sale, then these scores would go down (appear closer to random). For example, in Iowa or Illinois, one reason the portfolio covers so little of those states is that virtually the entire states have already been converted to farmland or urban areas. The data could be re-analyzed for 2001 to 2006, and the data could be obtained for each time period to be used to establish a range of natural land cover and public land coverage. However, complicating this analysis is the fact that in many cases TNC lands were transferred to public ownership, or public lands were transferred to TNC (typically as “trade lands,” but we have also outright purchased federal land to add to a preserve) [personal communication, 4/17/2012]. Also, while TNC usually purchases unconverted land, in some cases we do procure land converted to agriculture (or forest plantations) and restore it. The data would have to be carefully examined to ensure that not all public or converted land was excluded, but a “likelihood of unavailability” was incorporated somehow.

Second, by looking solely at area, we ignore contiguity and connectivity. In other words, our analysis considers a purchase entirely within a priority area as highly correlated, whereas a larger purchase that also extends outside of the priority area would show up as less correlated. However, in most cases the opportunity to purchase a large contiguous tract that extends outside of priority area boundaries would still be desirable, so an alternate approach could be to look at the percentage of lands that overlap the portfolio by at least some threshold (maybe 75%), and count any tract that overlaps the portfolio by that much as “aligned.”

Third, it has been established that it is difficult to determine whether or not defining priority areas has affected buying land in part because in some cases priority areas were defined around existing TNC lands. To better account for this, we could look for ecoregions where existing TNC lands were not considered in creating the portfolio, and split purchases into two time periods: before and after the creation of the portfolio. However, much of the information that went into the ecoregional assessments is unavailable or very difficult to locate, so for now this has simply been flagged as an area of future research.

**Table 4**. Results by time period for the 14 states with the most complete data (Arkansas, Georgia, Illinois, Indiana, Iowa, Kansas, Kentucky, Michigan, Minnesota, Mississippi, Missouri, Ohio, Tennessee, and Washington).

| **Time Period** | **Total Land Acquired, km^2^** | **Proportion of Acquisition Area in Portfolio** | **Science Influence Score** |
| --- | --- | --- | --- |
| **All Time** | **1,990.0** | **83.8%** | **74.5%** |
| 2006-2011 | 478.3 | 83.6% | 74.2% |
| 2000-2005 | 718.0 | 79.5% | 67.8% |
| Pre-2000 | 774.0 | 88.2% | 81.4% |
